# *Escherichia coli* aggravates inflammatory response in mice oral mucositis through regulating Th17/Treg imbalance

**DOI:** 10.3389/fcimb.2025.1585020

**Published:** 2025-04-29

**Authors:** Jia Liu, Wenhui Xia, Juehua Cheng, Yanlin Geng, Weiping Li, Yuan Fan

**Affiliations:** ^1^ Department of Oral Mucosal Diseases, The Affiliated Stomatological Hospital of Nanjing Medical University, Nanjing, China; ^2^ State Key Laboratory Cultivation Base of Research, Prevention and Treatment for Oral Diseases, Nanjing, China; ^3^ Jiangsu Province Engineering Research Center of Stomatological Translational Medicine, Nanjing, China; ^4^ Hefei Stomatological Hospital, Hefei Stomatology Clinical College of Anhui Medical University, Hefei, China

**Keywords:** oral mucosal immune inflammation, *Escherichia coli*, mice model, Th17/Treg response, mucosal immunity

## Abstract

**Introduction:**

Microbial dysbiosis links to mucosal immune dysregulation, but the specific bacterial contributions to oral mucosal inflammation remain unclear. *Escherichia coli* (*E. coli*), a pathogen well-characterized in mucosal immunity and immune regulation studies, has been observed to be enriched in chronic oral inflammatory lesions and was reported to modulate T helper 17 cells (Th17)/T regulatory cells (Treg) homeostasis. Here, we developed an oral mucositis mouse model via tongue scratch and *E. coli* topical application to investigate its role in Th17/Treg imbalance.

**Methods:**

The inflammatory infiltration was evaluated by macroscopic photography and HE staining. The expression of inflammatory factors in tongue tissue and peripheral blood of mice were detected by immunohistochemical staining and enzyme-linked immunosorbent assay. The number of Th17 and Treg in mice spleen lymphocytes were evaluated with flow cytometry. Differential gene expression analysis, functional enrichment analysis and immune infiltration analysis were performed using RNA-seq data from oral lichen planus (OLP).

**Results:**

*E. coli* stimulation aggravated inflammatory responses induced by scratching in lingual mucosa of mice, including increased local and systemic expression of interleukin 6 (IL6), interleukin 17 (IL17), chemokine receptor 6 (CCR6) and chemokine C-C motif ligand 20 (CCL20), increased proportions of Th17 cells and increased Th17/Treg ratio in spleen lymphocytes. Analysis of RNA-seq data from OLP revealed alterations in antimicrobial responses and inflammatory factors associated with upregulation of Th17/Treg balance.

**Conclusion:**

This study supports the role of *E. coli* in promoting oral mucosal inflammation and provides an experimental basis for *in vivo* study of OLP from the perspective of microorganisms.

## 1 Introduction

The human body harbors a collection of microorganisms and their genes and products, which settle in various sites such as gut, mouth, skin. These microorganisms help maintain immune system stability and fight off foreign pathogens (Korpela et al., 2020). Recent studies have shown that dysbiosis triggers abnormal immune responses and is a potential factor in the development of autoimmune diseases, including rheumatoid arthritis (Zhao et al., 2022; Xu et al., 2021; Lin et al., 2023), multiple sclerosis (Altieri et al., 2023; Thirion et al., 2023), inflammatory bowel disease (Qiu et al., 2022; Larabi et al., 2020; Gilliland et al., 2024), Sjogren’s syndrome (Cao et al., 2023; Zou et al., 2024), systemic lupus erythematosus (Yao et al., 2023). The oral microbiome is present in the unique microenvironment of the oral barrier and is the second largest microbial reservoir after the gut microbiome (Caselli et al., 2020). Advances in microbial sequencing technology have revealed that dysbiosis is associated with various oral mucosal diseases (Baker et al., 2024). For instance, *Malassezia* has been shown to dominate in active ulcers of recurrent aphthous ulcers (Stehlikova et al., 2019), while *Prevotella* and *Agrobacterium* are significantly enriched in pemphigus vulgaris (Li et al., 2023). Additionally, *Fusobacterium* abundance is notably increased in oral leukoplakia (Herreros-Pomares et al., 2023, 2021). The interaction of oral microbiota imbalance and inflammation may lead to the development of inflammatory diseases of the oral mucosa. Therefore, the complex relationship between dysbiosis and oral mucosal diseases has attracted intensive attention.

*Escherichia coli* (*E. coli*) is the world’s most studied microorganism and is usually found in the gastrointestinal tract of humans and animals (Geurtsen et al., 2022). In our previous study (Wang et al., 2020), 16S rDNA gene amplification sequencing and fluorescence *in situ* hybridization showed that *E. coli* was overexpressed in oral lichen planus tissues, which was consistent with the results of Keumjin Baek et al (Baek et al., 2020). Currently, *E. coli* is thought to have a potential pathogen-associated molecular pattern that mediates and exacerbates local immunoinflammatory responses by inducing high levels of interleukin-6 (IL-6) (Kittana et al., 2018). The role of *E. coli* in oral mucosal immunity deserves further exploration.

Th17 and Treg cells, which are important in building the body’s immune defenses, are differentiated from naïve CD4+ T cells (Knochelmann et al., 2018). Functionally, Th17 and Treg cells secrete different inflammatory cytokines. Notably, IL-6 plays a dominant role in mediating the differentiation of naïve T cells into Treg and Th17 cells (Starost et al., 2016). In addition, C-C motif chemokine receptor 6 (CCR6) and C-C motif chemokine ligand 20 (CCL20) are widely reported inflammatory markers beneficial to promote the recruitment of these two types of cells. The CCR6-CCL20 interaction may down-regulate forkhead box protein P3 (Foxp3) expression and enhance the expression of retinoic acid related orphan nuclear receptor gamma t (RORγt) and interleukin-17 (IL-17), transforming some Treg cells into pathogenic Th17 cells (Paldor et al., 2022; Nguyen et al., 2021). Interestingly, *E. coli* induces a pro-inflammatory response in epithelial cells via promoting the secretion of CCL20, and may alter the differentiation of Th17 and Treg cells (Paroni et al., 2023; Schirmer et al., 2019).

At present, *E. coli* is widely used in animal experiments related to inflammation such as meningitis (Yang et al., 2024), endometritis (Lindsay et al., 2023), enteritis models (Lin et al., 2019). However, in the context of oral mucosal inflammation, most existing animal models rely on chemoradiotherapy induction, which may not fully replicate the complex immune and microbial interactions observed in human diseases. While Candida albicans (C. albicans) infection models have been extensively studied for oral fungal infections (Liang et al., 2023), there remains a significant gap in modeling oral mucosal inflammatory diseases associated with immune disorders of unknown etiology, such as oral lichen planus. Further development of bacteria-induced models of oral mucosal inflammation may be beneficial to study the role of microorganisms in the occurrence and development of oral mucosal inflammatory diseases. In this study, we exploit a novel mouse model of oral mucosal inflammation using *E. coli* and investigate its effects on immune environment to provide an experimental basis for the *in vivo* study of oral immunoinflammatory diseases.

## 2 Methods

### 2.1 Bacterial culture

*E. coli* standard culture (ATCC 25922) was purchased from the American Model Culture Collection (ATCC). *E. coli* was cultured in LB broth at 37°C, 200 rpm with shaking overnight and transferred at a ratio of 1:100 the next day, and the optical density(OD) value was determined at 600nm by UV spectrophotometer 4h later.

### 2.2 Experimental animal and model construction

#### 2.2.1 Experimental mice

SPF grade 6–8 week male C57BL/6J mice provided by the Production Department of Pharmaceutical Laboratory Animal Center of Nanjing Medical University were selected and fed for 1 week at room temperature (23 ± 2) °C, humidity 40% ~ 70%, and diurnal environment, maintaining a pathogen-free state and eating and drinking freely. All methods of animal experiment study were approved by the Laboratory Animal Welfare Ethics Committee of Nanjing Medical University, and in accordance with the relevant provisions of the national laboratory animal welfare ethics. All animal experiments were in compliance with the ARRIVE guidelines (https://arriveguidelines.org).

#### 2.2.2 Specific methods for the animal model

Regarding the strategy of *E. coli* application in inducing inflammation of the tongue mucosa in mice, we considered two modalities in preliminary experiment: direct injection of the bacterial solution or smearing the bacterial solution after scratching. We found that the former way leaded to death, so we chose the latter option. After concentration screening, the final concentration of the bacterial liquid used in this study was 1 × 10^9^ CFU/mL (**Supplementary Figure S1**).

A total of 120 mice were divided into four groups by random number method: control group (n = 30), smeared group (n = 30), scratched group (n = 30), and scratched + smeared group (n = 30). All mice were anesthetized with 50 μL of chlorpromazine (2 g/L) intramuscularly in their thighs and kept them sedated for about 3 h. After anesthetizing the mice, sterile small forceps were used to gently pull the mouse tongue to the outside of the mouth, and the dorsal of the tongue in each group was treated as follows:

1. Control group: 50 μL normal saline was dipped in a sterilized cotton swab and evenly applied to the tongue mucosa of mice.
2. Smeared group: 50 μL 1 × 10^9^ CFU/mL *E. coli* suspension was dipped in a disinfectant cotton swab and evenly applied to the mouse tongue mucosa.
3. Scratched group: use a sterile 1 ml injection needle to dip an appropriate amount of gentian violet on the dorsal tongue of the mouse to locate it, and the scratch length is about 2–3 mm. Then take an additional sterile 1 mL injection needle and scratch the lingual mucosa (about 2–3 mm in length and 1–2 mm in depth) with the needle beveled upwards. After gently pressing the hemostasis with a sterile cotton swab, 50 μL of normal saline was applied to the scratched tongue mucosa.
4. Scratched + smeared group: use a sterile 1 ml syringe needle to scratch the tongue mucosa, and then dip 50 μL 1 × 10^9^ CFU/mL *E. coli* suspension with a sterile cotton swab to evenly apply the scratched tongue mucosa.

The mice in the smeared group and the scratched + smeared group were smeared 50 μL 1 × 10^9^ CFU/mL *E. coli* suspension once a day, while those in the control group or the scratched group were smeared equal volume normal saline. The general conditions of the mice were recorded. Ten mice were randomly selected from each group for observation on day 3, 5, and 7, respectively. After being photographed, the tongues were fixed in 4% paraformaldehyde for 24 hours.

To quantify the macroscopic manifestations, the severity of tongue lesions in mice was scored referring to the scoring systems that are widely used and validated in oral mucosal diseases, such as the ODSS, REU systems (Ormond et al., 2022; Unnikrishnan et al., 2023). Exactly, the lesions at the tongue modeling sites are scored on a 1–3 points range for the activity score: 0 for no erythema, no erosion or ulcer; 1 for mild erythema; 2 for obvious erythema without ulceration; 3 for erosion or ulcer. Besides, according to the overall situation of the mouse (**Supplementary Table S1**), the pain level was scored on a scale of 0–2 points with reference to the mouse grimace scale scores (Mogil et al., 2020). The activity score and pain score were finally summed to obtain the total score.

### 2.3 HE staining

The 4 μm tissue slices were heated at 65°C for 1 h, then treated with xylene for 1 min twice, followed by 1 min absolute ethanol twice, 95% ethanol twice, and 80% ethanol. After rinsing, the slices were stained with hematoxylin for 8 min, then treated with 0.5% hydrochloric acid ethanol for 1 s. Lithium carbonate for 1 min. After rinsing, 0.5% eosin solution for 10 s, then 80% ethanol, 95% ethanol twice, and absolute ethanol twice for 1 min each, followed by xylene for 3 minutes twice, and finally mounted. The H&E-stained tissue sections were measured using ImageJ (NIH, USA). The scale was calibrated using the embedded scale bar. The ulcer region was outlined with the freehand tool. The area of inflammatory cell infiltration surrounding the ulcer was quantified by thresholding and area measurement tools. The ulcer depth was measured as the vertical distance from the ulcer base to the adjacent intact epithelium using the straight-line tool.

### 2.4 Immunohistochemical staining and image analysis

Mouse tongue mucosal tissue sections were placed on anti-detachment slides and heated at 65°C to melt the wax. After deparaffinization with xylene twice, the sections were rehydrated through a gradient of alcohol (75%, 80%, 95%, 100%). After rinsing, antigen retrieval was performed by boiling in citric acid buffer (pH = 6.0). Following cooling and rinsing, a 3% hydrogen peroxide solution was applied to block catalase activity for 10 min, then rinsed and blocked with goat serum for 15 min. After spinning and wiping off the water, 50 μL of IL-6 antibody (1:100, Proteintech, USA), IL-17 antibody (1:1000, Proteintech, USA), CCR6 antibody (1:100, Proteintech, USA), and CCL20 antibody (1:100, Proteintech, USA) were added dropwise on the surface and incubated for 2–3 h at room temperature. The sections were rinsed and dried, then incubated with secondary antibody (MaxVision™ kit) for 15 min at room temperature. After rinsing, excess water was removed, and DAB was added for color development. Counterstained with hematoxylin, rinsed, and differentiated in 1% hydrochloric acid ethanol for 1–2 s, then returned to blue with lithium carbonate for 3–5 s. Dehydrated through a gradient of alcohol (75%, 80%, 95%, 100%) for 5 min. Cleared with xylene for 10 min twice and sealed.

The sections were scanned on an OLYMPUS VS200 whole slide scanning system. The positive area value, representing the product of the stained area and its optical density (OD), was measured using ImageJ software to quantify immunohistochemical staining and normalized to the control group.

### 2.5 Mouse peripheral blood collection and serum isolation

After anesthetizing, ophthalmic scissor was used to cut off the mice whiskers. Then the eyeball was removed quickly with forceps and collected the blood using a 1.5 ml EP tube. The mice were sacrificed by the cervical dislocation method. The peripheral blood samples were stored at 4°C for 3–4 hours, centrifuged at 3500 rpm for 10 min and the upper layer was aspirate gently using a sterile pipette. The extracted serum samples were stored at -80°C for further experiments.

### 2.6 Extraction of mouse spleen lymphocytes

The sacrificed mice were soaked in 75% ethanol for 3 to 5 min. the mouse spleen was transfer to a clean bench using sterile instruments, then the spleen tissues were trimmed to remove non-specific tissues (e.g., fat) and placed in Hanks’ solution. Then a 70 μm cell strainer was placed on a 50 mL centrifuge tube, and the spleen was transferred to the strainer moistened with Hanks’ solution. The spleen was gently ground using the piston of a 5 mL syringe. During grinding, Hanks’ solution was continuously added to rinse the tissue until the process was completed. Spleen cells were isolated using Mouse 1× Lymphocyte Separation Medium (Cat#: DKW33-R0100/DKW33-R0400). The cells were resuspended in 4 ml of RPMI-1640 medium. A 10 μl aliquot of cell suspension was mixed with 10 μL of trypan blue solution and cell counting was performed using a hemocytometer.

### 2.7 Flow cytometry

The isolated mouse spleen lymphocytes were centrifuged at 1000 rpm for 5 minutes. The supernatant was discarded, and the cells were washed with PBS. For surface staining, the following human monoclonal antibodies were used according to the manufacturer’s instructions: PerCP-CD3 (Biolegend, USA), FITC-CD4 (Biolegend, USA), APC-CD25 (Biolegend, USA), PE-CD127 (Biolegend, USA), PE-Cy7-CD183 (Biolegend, USA), PB450-CD196 (Biolegend, USA). The samples were then incubated at room temperature in the dark for 20 minutes, followed by washing with PBS and centrifugation at 1000 rpm for 5 minutes. After centrifugation, the supernatant was discarded, and the cell pellet was resuspended in 300 μL of PBS. The samples were analyzed using CytExpert software (Treestar, USA) within 1 hour.

### 2.8 Enzyme-linked immunosorbent assay

The detection of inflammatory cytokines and chemokines, including IL-6, IL-17, CCR6 and CCL20, was performed using commercially available enzyme-linked immunosorbent assay (ELISA) kits (Mlbio, China). Each sample was assayed in triplicate wells. After zeroing the plate reader using a blank well, the optical density (OD) values of each well were measured at a wavelength of 450 nm. The average OD values of the triplicate wells were calculated and analyzed using Curve Expert 1.4 software. A standard curve was generated, and the corresponding cytokine concentrations were determined based on the curve.

### 2.9 Transcriptomic analysis

To investigate the transcriptional distinction between intact and erosive states in oral mucosal immune-inflammatory diseases, we downloaded RNA sequencing data of OLP from the GSE213346 dataset from Gene Expression Omnibus (GEO, https://www.ncbi.nlm.nih.gov/geo/). This dataset included four groups of OLP patients categorized based on initial diagnosis and one-year follow-up outcomes (RE for recurrent erosive oral lichen planus follow-up, S for stable oral lichen planus follow-up): EOLP-RE, EOLP-S, NEOLP-RE, and NEOLP-S (Qing et al., 2023). To mitigate potential similarities arising from patient grouping within this dataset, we focused our analysis on EOLP-RE (n = 7) and NEOLP-S (n = 24), which were two clinically distinct phenotypes with clear divergence in symptom severity and disease progression. This contrast allows us to identify molecular signatures strongly associated with disease activity and prognosis. The read counts from each sequenced sample were combined into a count file, which was subsequently used for the differential expression analysis. Differential analyses were performed to the count files using the R package “DESeq2”, applying thresholds of an adjusted P < 0.05 and | log2 FC |> 1. The R package “clusterProfiler” was employed to perform Gene Ontology (GO) annotation and Kyoto Encyclopedia of Genes and Genomes (KEGG) enrichment analysis. The GO categories encompassed three sections: biological processes (BP), molecular functions (MF), and cellular components (CC), with a significance threshold set at an adjusted P-value<0.05 for the enrichment pathways (Yu et al., 2012). The gmt files of GO and KEGG were downloaded from GSEA website and were performed with “clusterProfier” package with a significance threshold set at an adjusted P-value<0.05 for the enrichment pathways. To evaluate the immune infiltration among different samples, the CIBERSORT algorithm was used to estimate the abundance of 22 distinct immune cells based on gene expression profiles (Newman et al., 2015). The data were visualized using the “ggplot2”.

### 2.10 Statistical analysis

GraphPad Prism software (GraphPad, v.9.4.1) was used for the statistical analyses. Data are represented as the means ± SD. Student’s t-test was applied to compare the results between two sample groups if the data were normally distributed. One-way analysis of variance (ANOVA) was used to compare means across three groups, with multiple comparisons adjusted using either the Dunnett’s or Tukey’s test.

## 3 Results

### 3.1 Scratching plus *E. coli* smearing induced tongue mucositis in mice

**Figure 1:**
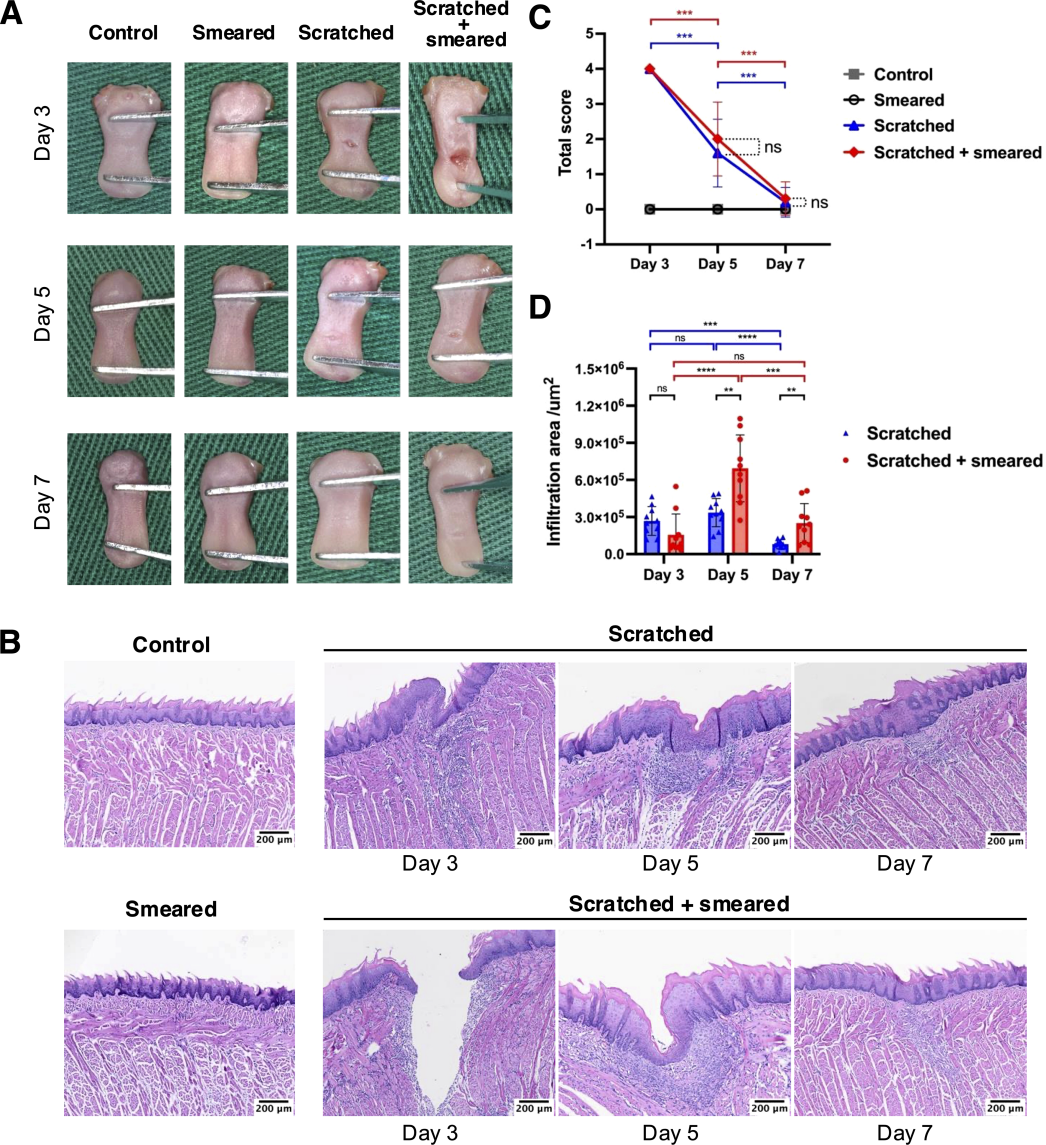
Macroscopic manifestations and HE staining of tongue tissues of mice. **(A)** Macrophotography of mice tongue tissues on days 3/5/7. **(B)** HE staining results of mice tongue tissues in different groups. Scale bar: 100 μm. **(C)** The total clinical symptom scores of the mice tongue tissue to assess the severity of the modeling lesions(n = 10/group/day). The asterisk (*) indicates statistical significance by Student's t test. *** *p* < 0.001, ns, no significance. **(D)** The infiltration area of inflammatory cells at the lesion sites from the mice tongue tissues based on HE staining results (n = 10/group/day). The asterisk (*) indicates statistical significance by Student's t test. ** *p* < 0.01, *** *p* < 0.001, **** *p* < 0.0001, ns, no significance.

The mice in the scratched group and the scratched + smeared group showed an inflammatory response on day 3, including ulceration, exudation, and congestion at the tongue site (**Figure 1A**). On days 5 to 7, the wound contraction decreased, and the wound gradually healed. However, the tongues of mice that were only smeared remained in the same state as those of the control group. The scratched + smeared group and the scratched group received the highest score on day 3, and showed a statistically significant decrease on days 5 and 7 (*p* < 0.001) (**Figure 1C**).

In order to investigate the local inflammation alteration, we performed HE staining chiefly on tongue tissues of the scratched group and the scratched + smeared group collected on days 3, 5, and 7. The results of HE staining showed increased degrees of local inflammation in the scratched and scratched + smeared group, while the smeared group and the control group showed no significant inflammatory response, consistent with the macroscopic manifestations (**Figure 1B**). Microscopically, both the scratched group and the scratched + smeared group had noticeable ulcer and destruction of the epithelial layer on day 3, accompanied by local fiber exudation, and inflammatory cells began to accumulate in the lamina propria. To compare the degree of inflammation between the scratched group and the scratched + smeared group, we evaluated the area of inflammatory cell infiltration at the site of modeling (**Figure 1D**). The inflammatory cell infiltration area of the scratched + smeared group reached the highest level on day 5, and there was a statistically significant difference compared with day 3 (*p <*0.0001) or day 7 (*p* < 0.001). Meanwhile, on day 5, the area in the scratched + smeared group was significantly larger than that in the scratched group (*p* < 0.01). On day 3, the inflammatory infiltration area of the scratched + smeared group was lower than that of the scratched group, although it was not statistically significant (*p* > 0.05), which may be due to the larger ulcer depth in the scratched + smeared group and the local inflammatory response not reaching its peak (**Supplementary Figure S2**).

Notably, there was no obvious inflammatory response in the mice in the smeared group, both in general and under the microscope. Thus, in the following part, we conducted further experiments on the mice in the control group, the scratched group, and the scratched + smeared group.

### 3.2 Inflammatory cytokines and chemokines were upregulated in mice tongues treated with scratching plus *E. coli* smearing

**Figure 2:**
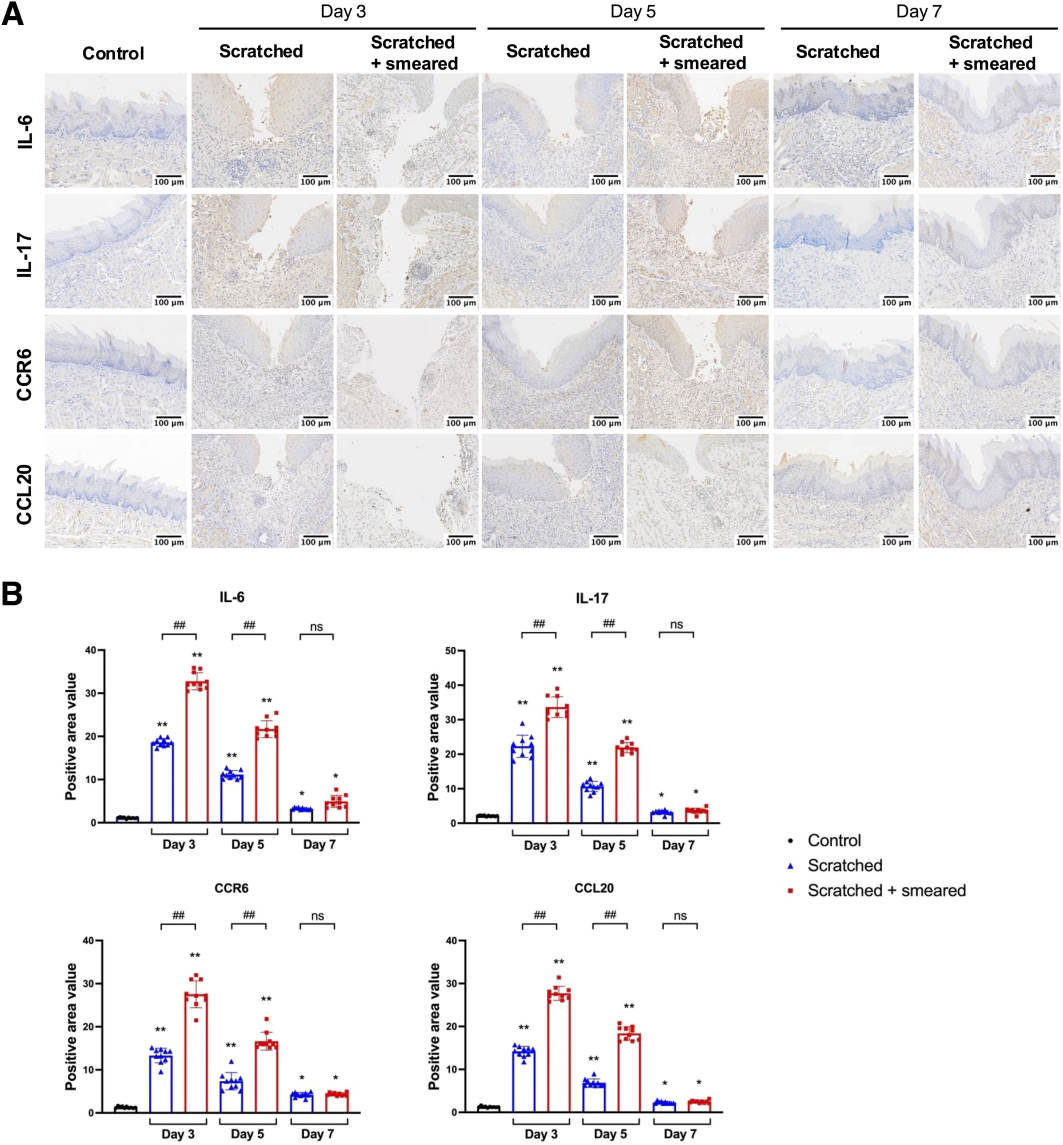
Expression of IL-6, IL-17, CCR6 and CCL20 in the tongue mucosa of mice. **(A)** Representative immunohistochemical image of IL-6, IL-17, CCR6 and CCL20 expression in the tongue tissues of mice in the control group, the scratched group, and the scratched + smeared group. Scale bar: 50 μm. **(B)** Statistical analysis of expression of IL-6, IL-17, CCR6 and CCL20 in the tongue tissues of mice in the control group, the scratched group, and the scratched + smeared group (n = 10/group/day). Asterisks (*) represent significant differences versus the control group; hash symbols (#) represent significant differences between the scratched group and the scratched + smeared group (Student’s *t* test, * *p* < 0.05, ** *p* < 0.01, ## *p* < 0.01, ns, no significance).

We performed immunohistochemical staining to detect expression of inflammatory factors in mice tongues after scratching and topical application of *E. coli*. The positive expressions of IL-6, IL-17, CCR6 and CCL20 were observed in the local inflammatory cells of the subepithelial lamina propria of the tongue mucosa in the experimental group (**Figure 2A**). In the tongue mucosa of mice, the expression levels of IL-6, IL-17, CCR6, and CCL20 were significantly higher in the scratched group and scratched + smeared group than in the control group. The increases were most pronounced on days 3 and 5 (*p* < 0.01), with a slight attenuation by day 7 (*p* < 0.05). In addition, the expressions of IL-6, IL-17, CCR6 and CCL20 in the scratched + smeared group were significantly higher than those in the scratched group on day 3 and day 5 (*p* < 0.01), while there was no significant difference between the two groups on day 7 (**Figure 2B**).

### 3.3 Expression levels of systemic inflammatory factors in mice with oral mucositis

**Figure 3:**
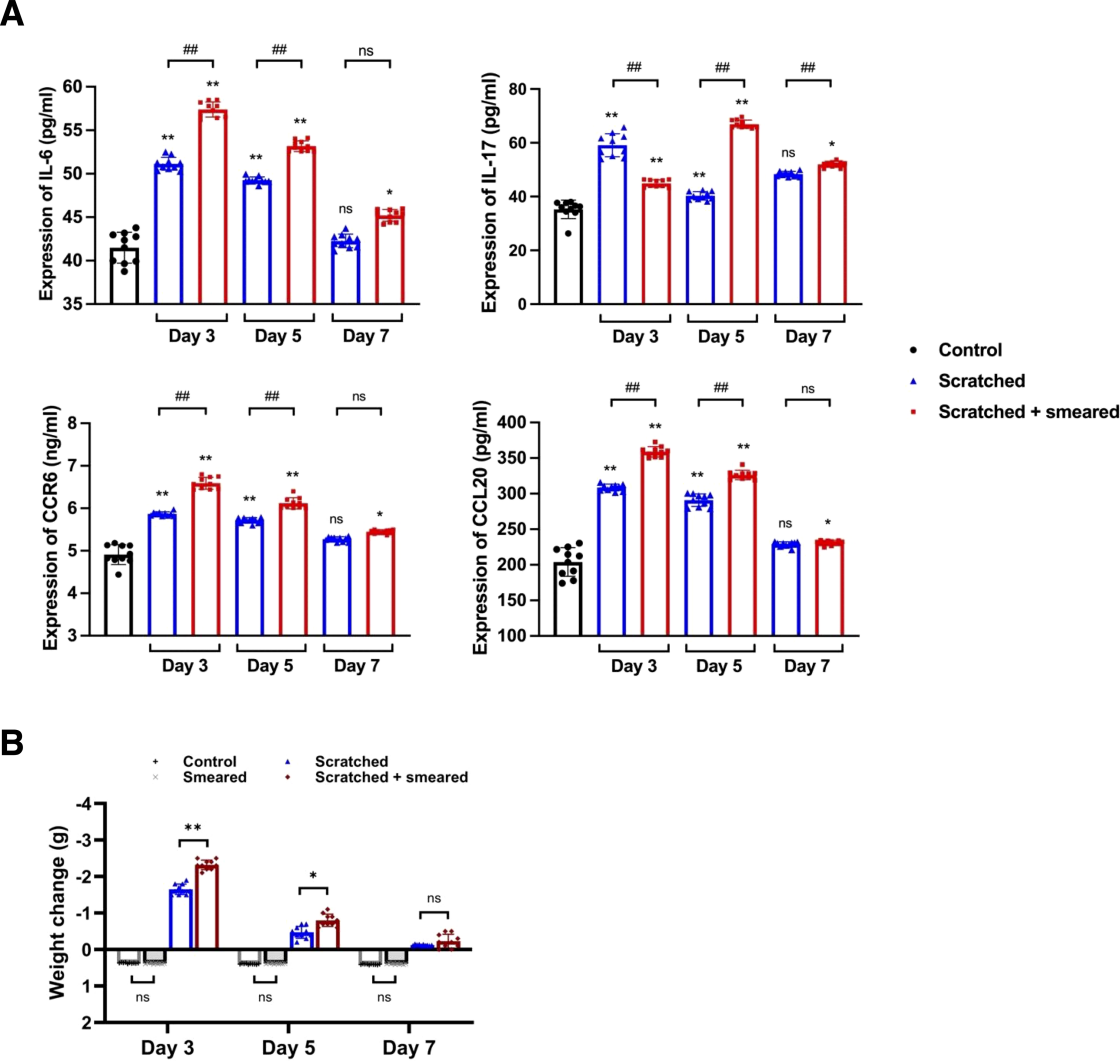
Typical inflammatory factors were upregulated in mice treated with scratching and *E. coli* smearing. **(A)** ELISA was used to detect the expression of IL-6, IL-17, CCR6 and CCL20 in peripheral blood serum samples of mice (n = 10/group/day). Asterisks (*) represent significant differences versus the control group; hash symbols (#) represent significant differences between the scratched group and the scratched + smeared group (Student’s *t* test, * *p* < 0.05, ** *p* < 0.01, ## *p* < 0.01, ns, no significance). **(B)** The body weight changes of mice on days 3/5/7 during modeling. The body weight of the control group and the smeared group increased, while the scratched group and the scratched + smeared group decreased. Data present the individual values and mean with SD of each group. Student’s *t* tests were conducted between the control group and the smeared group, as well as between the scratched group and the scratched + smeared group (n = 10/group/day). * *p* < 0.05, ** *p* < 0.01, ns, no significance.

To assess the levels of Th17/Treg-related inflammatory factors in mice, we measured the protein expression of IL-6, IL-17, CCR6 and CCL20 in the serum of mice by ELISA. The results showed the expressions of IL-6, IL-17, CCR6 and CCL20 in the peripheral blood serum of mice in the scratched group and the scratched + smeared group on day 3 and day 5 were significantly higher than those in the control group (*p* < 0.01) (**Figure 3A**). The expressions of IL-6, IL-17, CCR6 and CCL20 in the peripheral blood serum of mice of the scratched +smeared group were significantly higher than those in the scratched group on day 3 and day 5 (*p* < 0.01) (**Figure 3A**).

Given the upregulation of systemic inflammatory factor levels in mice, we noted changes in mouse body weight during modeling, which may also be a manifestation of systemic responses in mice. The weight of the control group and the smeared group increased slowly on day 3, 5, and 7 after modeling (**Figure 3B**), and there was no significant difference in weight gain (*p* > 0.05). The weight of the scratched group and the scratched + smeared group decreased on days 3, 5, and 7 after modeling. The weight losses of the scratched group and the scratched + smeared group were the largest on day 3, while the scratched + smeared group was more significant (*p* < 0.01). On day 5, the weight losses of the scratched group and the scratched + smeared group decreased, but the weight loss of the scratched + smeared group was still more significant than that of the scratched group (*p* < 0.05). Weight recovered on day 7, and there was no significant difference between the two groups (*p* > 0.05).

### 3.4 The ratio of Th17/Treg cells were upregulated in the scratched + *E. coli* smeared group

**Figure 4:**
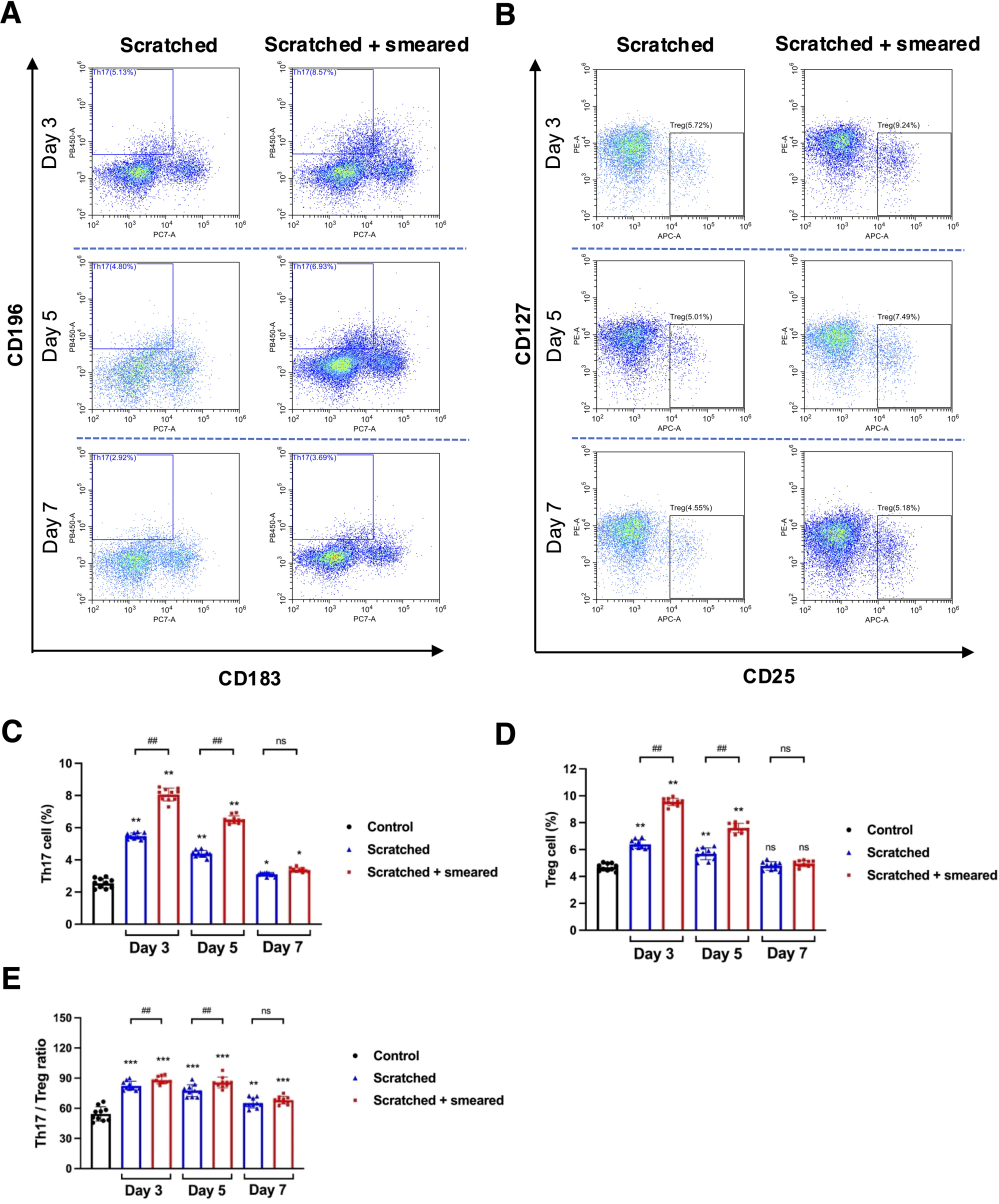
The ratio of Th17/Treg cells were upregulated in the scratched + *E. coli* smeared group. **(A)** Representative image of flow cytometry of Th17 cells from the spleen lymphocytes in the scratched group and the scratched + smeared group. **(B)** Representative image of flow cytometry of Treg cells from the spleen lymphocytes in the scratched group and the scratched + smeared group. **(C, D)** The proportion of Th17 and Treg cells from spleen lymphocytes of the scratched group and the scratched + smeared group (n=10/group/day). **(E)** The Th17/Treg ratio of the scratched group and the scratched + smeared group (n=10/group/day). Asterisks (*) in **(C-E)** represent significant differences versus the control group; hash symbols (#) in **(C-E)** represent significant differences between the scratched group and the scratched + smeared group (Student’s t test, * *p* < 0.05, ** *p* < 0.01, # *p* < 0.05, ## *p* < 0.01, ns, no significance).

To understand the effect of inflammation caused by oral mucosal trauma and infection on the Th17/Treg balance of the immune system, we assessed the number of Th17 and Treg cells in mouse spleen lymphocytes using flow cytometry. The CD3+CD4+CD196+CD183- cells were labeled as Th17 cells (**Figure 4A**), and the CD3+CD4+CD25+CD127- cells were labeled as Treg cells (**Figure 4B**). The flow cytometry results showed that the proportions of Th17 and Treg cells and the Th17/Treg ratio of the scratched group and the scratched + smeared group on day 3 and day 5 were significantly higher than those of the control group (*p* < 0.01) (**Figures 4C–E**). Compared with the scratched group, the proportion of Th17 and Treg cells and the ratio of Th17/Treg on day 3 and day 5 were significantly higher in the scratched +smeared group (*p* < 0.01) (**Figures 4C–E**).

### 3.5 RNA signature of EOLP characterized by upregulation of antimicrobial response and inflammatory factors

The integrity of the epithelial barrier plays a crucial role in maintaining mucosal immune homeostasis. In animal experiments, we observed that mechanical injury significantly facilitated microbial invasion into the mucosa and induced inflammatory infiltration, suggesting that the disruption of the oral mucosal barrier may contribute to the development and exacerbation of diseases (such as OLP), potentially leading to diverse clinical manifestations and prognoses.

**Figure 5:**
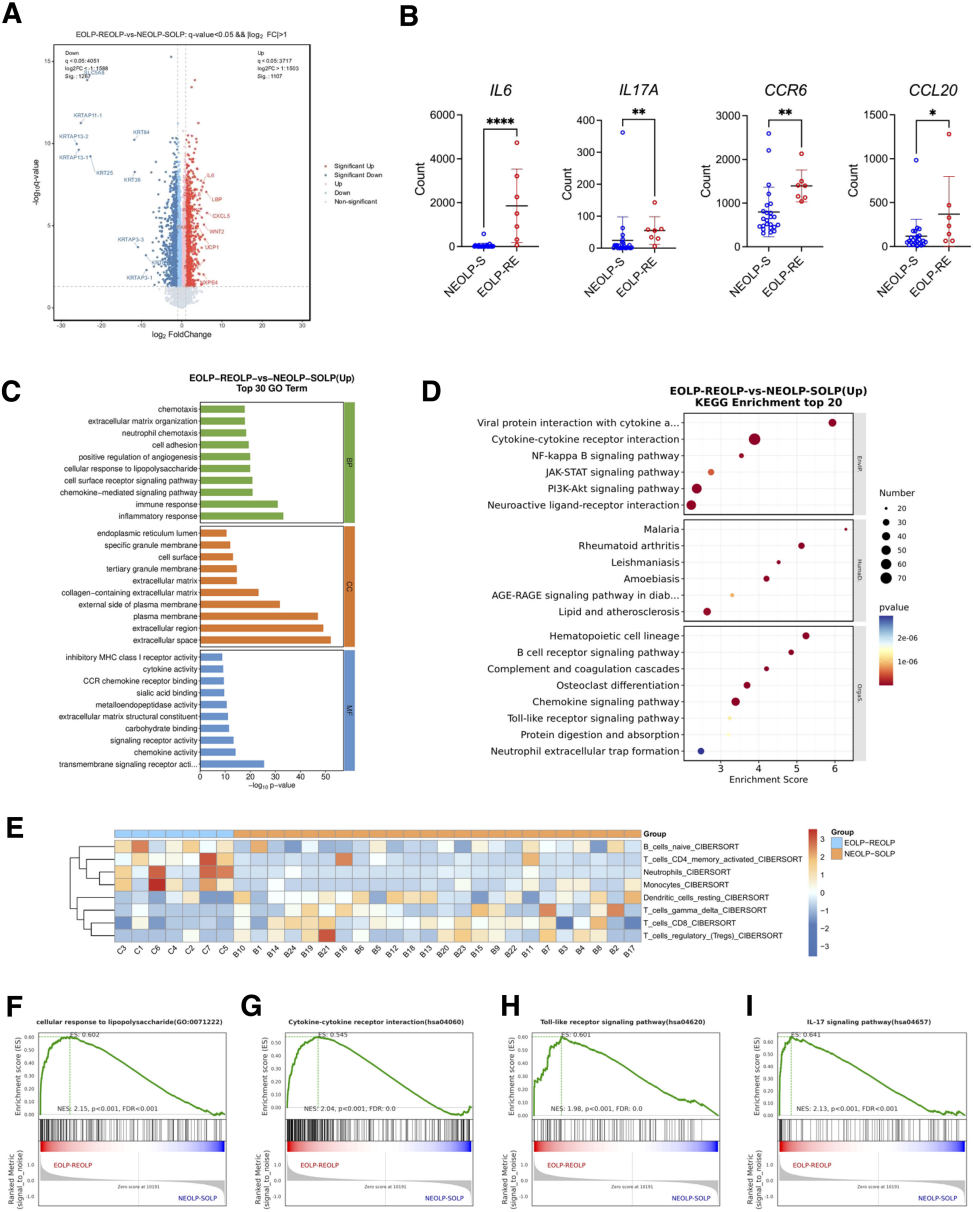
RNA signature of EOLP characterized by upregulation of antimicrobial response and inflammatory factors. **(A)** Volcano plots showed differential expression genes between EOLP-RE group and NEOLP-S group. **(B)** Count number of *IL6*, *IL17A*, *CCR6*, and *CCL20* between NEOLP-S (n = 24) and EOLP-RE groups (n = 7) in RNA-seq data. *p < 0.05, **p < 0.01, ****p < 0.0001. **(C)** Results of Gene Ontology (GO) enrichment analysis. **(D)** Results of KEGG pathway analysis. **(E)** Heat map showed immune infiltration scores between NEOLP-S and EOLP-RE samples based on CIBERSORT. **(F-I)** Results of Gene Set Enrichment Analysis. (EOLP-RE, erosive oral lichen planus with recurrent erosive oral lichen planus follow-up, NEOLP-S, non-erosive oral lichen planus with stable oral lichen planus follow-up).

Differential expression analysis between EOLP-RE and NEOLP-S groups revealed that the RNA expression levels of inflammatory factors such as *IL6*, *CXCL5*, and *CCL20* were significantly upregulated in EOLP-RE (*p* < 0.05) (**Figure 5A**). Specifically, we investigated several cytokines of interest from animal experiments and found that the expression of *IL6*, *IL17A*, *CCR6*, and *CCL20* was significantly higher in EOLP-RE compared to NEOLP-S (**Figure 5B**). To understand the biological functions of the differentially expressed genes, we performed GO enrichment analysis and KEGG enrichment analysis based on upregulated differential genes. The GO enrichment results indicated significant enrichment of biological processes such as immune response, inflammatory response, and cellular response to lipopolysaccharide among the upregulated genes. In terms of molecular function, significant enrichment was observed in signaling receptor activity, chemokine activity, and CCR chemokine receptor binding (**Figure 5C**). Similarly, KEGG pathway analysis highlighted enrichment in cytokine-cytokine receptor interaction, NF-kappa B signaling pathway, and chemokine signaling pathway, suggesting a robust immune-inflammatory response in EOLP (**Figure 5D**). Immune infiltration analysis based on CIBERSORT showed that the scores of neutrophils and activated CD4+ memory T cells were elevated in EOLP-RE compared with NEOLP-S group (**Figure 5E**). Immune cells, including activated CD4+ memory T cells, neutrophils, monocytes and B cells, were significantly upregulated in the EOLP-RE group compared to the NEOLP-S group(**Supplementary Figure S3**). Additionally, we performed GSEA and the TOP30 enrichment results showed that cellular response to lipopolysaccharide, cytokine-cytokine receptor interaction, the toll-like receptor signaling pathway and IL-17 signaling pathway were also significantly upregulated in EOLP-RE group (**Figures 5F–I**). These bioinformatic findings were in line with our above experimental results, suggesting that the colonization and invasion of gram-negative bacteria such as *E. coli* may contribute to the intensive inflammatory response in OLP lesions.

## 4 Discussion

In this study, we constructed a mouse model of tongue mucositis by mechanically scratching the tongue mucosa followed by topical application of *E. coli*, and we evaluated it by histological and bioinformatic analysis. The present data revealed that scratching plus *E. coli* smearing increased the expression of inflammatory cytokines IL-6 and IL-17 and chemokines CCL20 and CCR6, exacerbating the imbalance between Th17 and Treg, thereby aggravating the local immune inflammatory response similar with OLP.

The oral mucosal barrier serves as the first line of defense against various external stimuli, responding to physicochemical damage, microorganisms and their metabolites, as well as the host’s immune status (Moutsopoulos and Konkel, 2018; Williams et al., 2021). Physicochemical stimulus are commonly used in the study of animal models of mucosal diseases. In models of gastric ulcer, methods such as ethanol, ischemia-reperfusion, and ibuprofen treatment were used to induce lesions in rats (Araújo et al., 2024). Mechanical scratches combined with chemical stimulation have also been shown to be effective in mimicking the inflammatory response and healing process of chronic wounds in skin wound models (Jia et al., 2023). In our study, we aimed to investigate the potential of bacteria in the induction of oral mucosal inflammation model. Notably, topical application of *E. coli* lysate alone failed to induce macroscopic lesions in the murine oral mucosa. In contrast, mechanical scratch-induced injuries on the tongue exhibited limited tissue damage and demonstrated rapid epithelial regeneration. This may be related to the special anatomy and strong healing ability of the oral mucosa of mice (Su et al., 2025), and its response mechanism to external stimuli may be different from that of other tissues (Seubert et al., 2024; Kusumoto et al., 2024).

In the past, the oral mucosal inflammation model was mainly induced by chemoradiotherapy for diseases such as oral cancer and chemoradiotherapy ulcers (Murphy et al., 2008; Jordan et al., 2021). However, there are still many oral mucosal states that lack *in vivo* experimental models, resulting in a lack of insight into the mechanisms of the disease (Choi, 2024). For lichenoid lesions of the oral mucosa, cell-mediated immune disturbances and inflammatory infiltrates are thought to be important pathogenesis (Vičić et al., 2023). Our bioinformatics analysis revealed distinct immune cell infiltration patterns between EOLP-RE and NEOLP-S groups, particularly the upregulation of activated CD4+ memory T cells, neutrophils, and B cells in EOLP-RE. This suggests that disruption of the mucosal barrier in erosive lesions may promote bacterial penetration, trigger innate immune cell recruitment, and exacerbate or maintain mucosal inflammation. In recent years, numerous studies have shown that bacterial infections play an important role in the occurrence and progression of a variety of diseases. For example, in inflammatory bowel disease, dysbiosis of the bacterial flora has been shown to be strongly associated with intestinal mucosal barrier damage and inflammatory response (Mirsepasi-Lauridsen et al., 2019). Similarly, in a chronic diabetic ulcer model, bacterial infection not only exacerbates local inflammation, but also delays wound healing (Chang and Nguyen, 2021). These suggest that bacterial infections may be one of the key factors in mimicking the pathological process of disease. Similarly, oral mucosal pathologies are characterized by microbiota dysbiosis, which demonstrates a significant etiopathogenic correlation with disease initiation and progression (Carvalho et al., 2019; Jung and Jang, 2022). Based on the immune dysregulation observed in EOLP-RE, future work could consider refining *in vivo* models targeting specific oral pathogens to explore therapeutic strategies to restore mucosal immune homeostasis. Previous studies have reported that *E. coli* is enriched in oral lichen planus, a chronic inflammatory disease, and may promote the development of inflammation by regulating Th17/Treg balance at the cellular level (Wang et al., 2023a; Zhang et al., 2023). In this study, we introduced *E. coli* infection into oral mucosal inflammation model, which was found to significantly enhance the inflammatory response and mimic the pathological features observed in clinical practice. This result further supports the importance of bacterial infections in the construction of oral mucosal disease models.

The balance of Th17/Treg cells plays a key role in the development of mucosal immunoinflammatory diseases (Wang et al., 2023b; Saviano et al., 2024). Th17 cells are able to secrete a variety of pro-inflammatory factors that enhance mucosal immunity against extracellular pathogens (Gaffen and Moutsopoulos, 2020), while Treg cells are mainly responsible for inhibiting the body’s excessive immune response (Plitas and Rudensky, 2016). IL-6 is a pleiotropic immunomodulator that promotes host defense by stimulating acute-phase responses, hematopoiesis, and immune responses in response to infection and tissue injury (Kang et al., 2020). IL-6 plays an important role in both innate and acquired immunity by promoting the differentiation of naïve CD4+ T cells into Th17 and inhibiting TGF-β-induced Treg differentiation (Rose-John et al., 2023). IL-17, as a pro-inflammatory factor, is essential in the host’s defense against mucosal infection, promoting tissue repair by maintaining the mucosal barrier and upregulating antimicrobial proteins (Vidal et al., 2021; Saran et al., 2025). Our results showed the high expression of IL-6 and IL-17 in the tongue mucosa and peripheral blood of scratched + smeared mice, suggesting that *E. coli* induces up-regulation of local and systemic inflammatory factors and influences Th17/Treg balance. Besides, mice in both scratched and scratched + smeared groups exhibited weight loss, likely due to pain-induced feeding suppression and inflammation-driven metabolic alterations (Peña-Durán et al., 2025). The weight loss in the scratched + smeared group suggests a synergistic effect of mechanical injury and bacterial challenge on systemic inflammation. In addition, *E. coli* exacerbated inflammation in the tongue mucosa of the scratched mice, with an increase in the number of Th17 and Treg cells and an increase in the Th17/Treg ratio in the spleen lymphocytes. The high expression of CCR6 and ligand CCL20 might drive the migration of Th17/Treg cells to CCL20-rich inflammatory tissues, promoting Th17/Treg-mediated inflammatory response (Nguyen et al., 2021; Shi et al., 2020).

Admittedly, animal model study of oral mucositis is inherently challenging and our study has some limitations. In the process of modeling exploration, we found that the oral mucositis of mice rarely showed obvious long-term ulcers or erosions, and the duration of oral mucosal inflammation in mice was short. We observed mice only for the duration of the disease and did not assess the long-term effects of modeling. In fact, in rodents, the appearance of oral ulcers is rare, and in many models there is only histological evidence of a decrease in epithelial thickness suggesting the occurrence of mucositis (Al-Azri et al., 2015). This may be due to the obvious differences in oral anatomy and physiology between rodents and humans. The thinner keratinized epithelium and low epithelial extension of rodents minimize transport across the mucosa and reduce their sensitivity to significant injury (Wardill et al., 2019). In addition, mice have strong vitality and strong self-repair ability. These differences affect the sensitivity of the rodent oral mucosa to allergens, toxins, and other pathogens, and reduce the clinical manifestations of inflammatory lesions of the mucosa (Thirion-Delalande et al., 2017). In addition, in order to control the individual differences in soft tissue modeling as much as possible, we used randomization, control of the concentration and volume of bacterial suspension, and labeling of the stain before scratching the tongue mucosa. Still, it may not be possible to avoid the differences inherent in mouse tongue mucosal tissue modeling. Clinically, the pathogenesis of oral mucosal immune disorders such as OLP is a multifactorial process. The stimulation of a single bacterium may not enough to completely replicate the disease manifestations, but it can still induce inflammation of the oral mucosa in the oral cavity of mice, including the increased expression of certain inflammatory factors. Future *in vivo* studies may integrate autoimmune responses and dysbiosis of the microbiota to mimic OLP.

Overall, our study showed that scratching plus *E. coli* smearing induced tongue mucositis in mice by regulating Th17/Treg balance. The colonization of *E. coli* in tongue mocusa trigger local inflammation, as well as systemic inflammatory responses. This new mice model of oral mucositis may shed light for *in vivo* experimental studies of inflammatory diseases of the oral mucosa in the perspective of microbiology.

## Data Availability

The datasets presented in this study can be found in online repositories. The names of the repository/repositories and accession number(s) can be found in the article/**Supplementary Material**.
