# Supplementary material for: Escherichia coli aggravates inflammatory response in mice oral mucositis through regulating Th17/Treg imbalance
Source: Front Cell Infect Microbiol. 2025 Apr 29;15:1585020. doi: 10.3389/fcimb.2025.1585020 (PMC12069327; doi:10.3389/fcimb.2025.1585020)
Supplement: Supplementary file 2 [file Table2.docx]

Supplementary Material

# Supplementary explanation of the method

## Selection of molding method and bacterial concentration

### Pre-experiment to choose molding method.

Scheme 1: Local injection of *E. coli* suspension. Three mice were randomly selected for local injection of *E. coli* suspension into the tongue mucosa, and it was unfortunately found that two mice died on the same day and one died the next day. Thus, this operation method was abandoned.

Scheme 2: The combination of mechanical damage and smearing *E. coli* suspension. Sterile dental probes and 1ml injection needles are two alternative scratching tools. Comparatively, the operation of scratching the tongue mucosa with a sterile injection needle is more implementable.

### Selecting *E. coli* concentration

According to the literature guidance of *E. coli*-related inflammation models (Lin et al., 2019) (Wei et al., 2023), three concentration gradients of 1 × 10^7^ CFU/mL, 1 × 10^8^ CFU/mL, and 1 × 10^9^ CFU/mL were selected to screen out the optimal *E. coli* inducible concentration. Twenty-seven mice were randomly selected and divided into three groups: scratch +1 × 10^7^ CFU/mL smear group (n = 9); scratch +1 × 10^8^ CFU/mL smear group (n = 9); scratch +1 × 10^9^ CFU/mL smear group (n = 9). Three mice in each group were randomly sacrificed on the days 3/5/7 to observe the inflammation of the tongue mucosa. It was found that 1 × 10^9^ CFU/mL group had the largest inflammatory response, so this concentration was selected for subsequent experiments.

**References**

LIN, Q., SU, G., WU, A., CHEN, D., YU, B., HUANG, Z., LUO, Y., MAO, X., ZHENG, P., YU, J., LUO, J. & HE, J. 2019. Bombyx mori gloverin A2 alleviates enterotoxigenic Escherichia coli-induced inflammation and intestinal mucosa disruption. *Antimicrob Resist Infect Control,* 8**,** 189.

WEI, J., CHEN, C., FENG, J., ZHOU, S., FENG, X., YANG, Z., LU, H., TAO, H., LI, L., XV, H., XUAN, J. & WANG, F. 2023. Muc2 mucin O-glycosylation interacts with enteropathogenic Escherichia coli to influence the development of ulcerative colitis based on the NF-kB signaling pathway. *J Transl Med,* 21**,** 793.

# Supplementary Figures


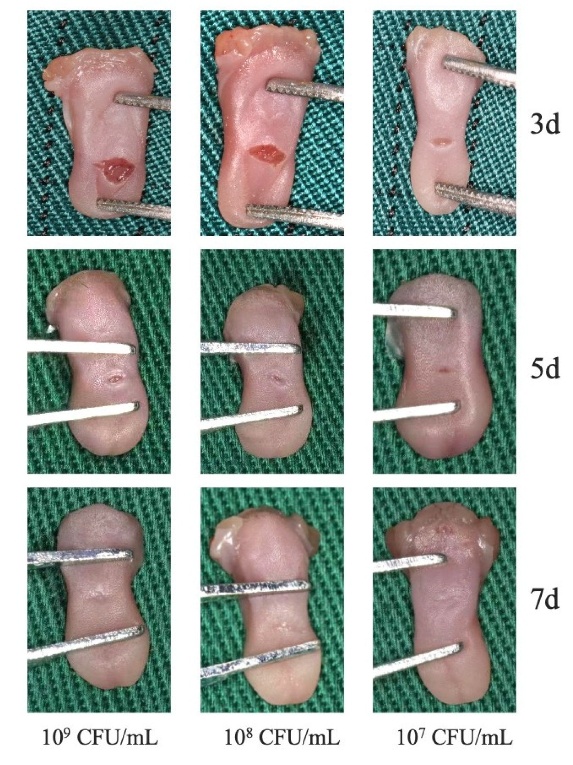


**Supplementary Figure 1.** Images of mice tongue mucosa on days 3/5/7 after being scratched and daily smeared with +1 × 10^7^ CFU/mL, 1 × 10^8^ CFU/mL, 1 × 10^9^ CFU/mL *E. coli* (scratched +smeared 1 × 10^7^ CFU/mL days 3/5/7: n = 3; scratched + smeared 1 × 10^8^ CFU/mL days 3/5/7: n = 3; scratched + smeared 1 × 10^9^ CFU/mL days 3/5/7: n = 3)


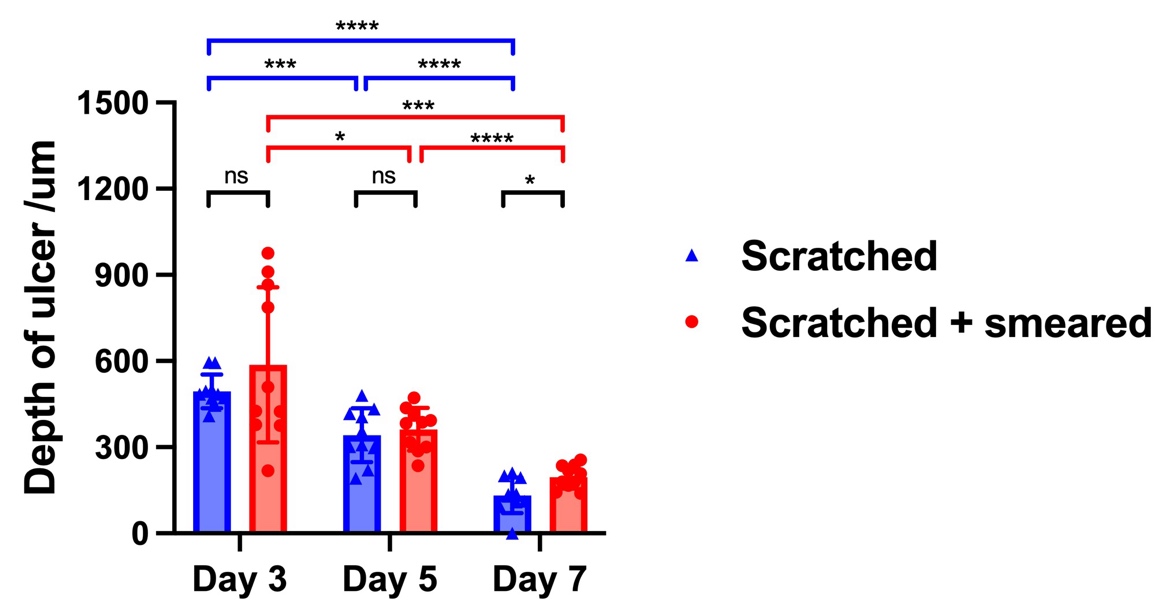


**Supplementary Figure 2.** Ulcer depth at the modeling site in HE staining. The Student’s *t* test was used to compare the differences between two groups. The blue lines show the comparisons of the scratched groups over time. The red lines show the comparisons of the scratched + smeared groups over time. The black lines show comparisons of the scratch group and the scratch group at the same time. **** *p* < 0.0001, *** *p* < 0.001, * *p* < 0.05, ns, no significance.

**
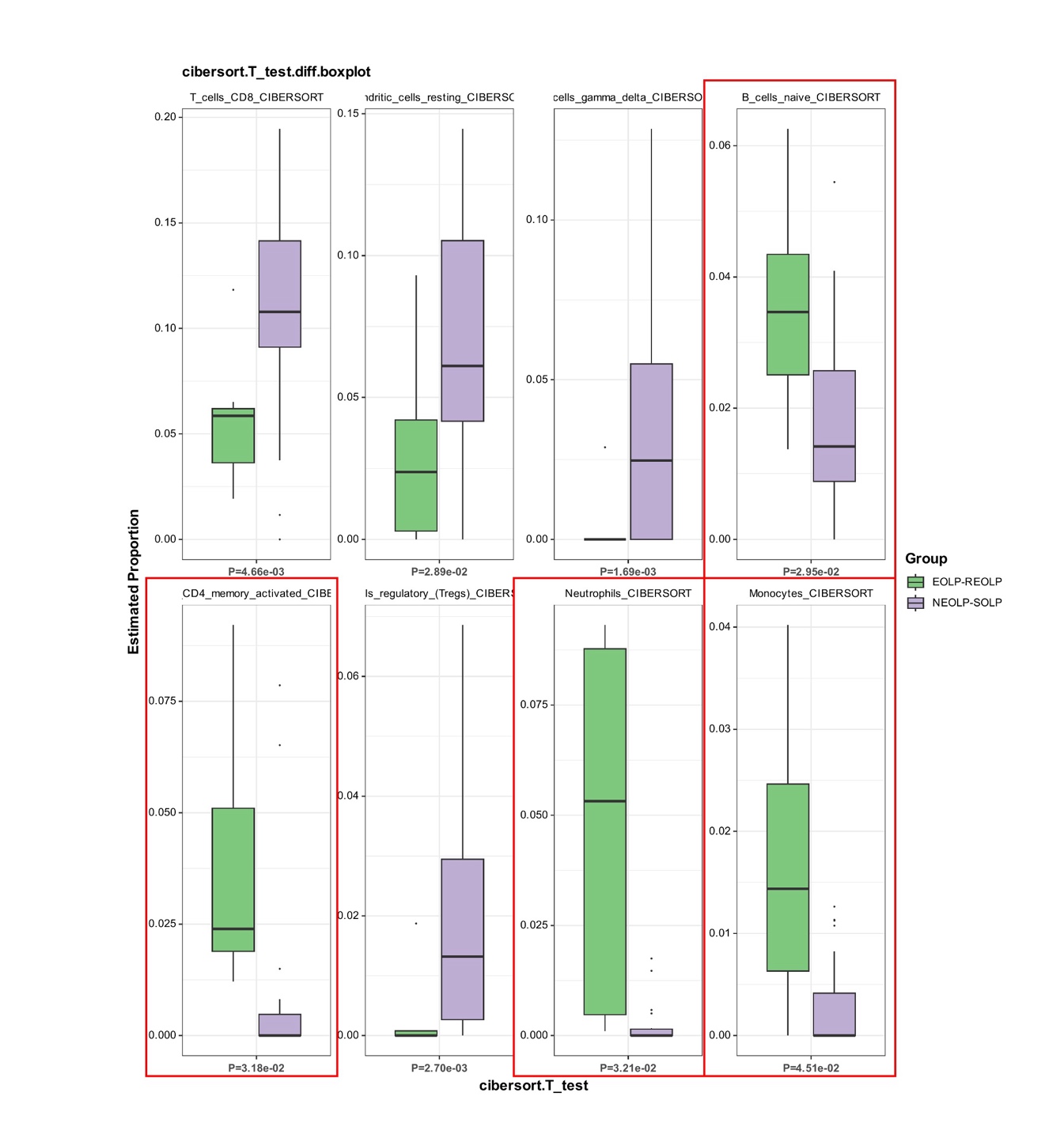
**

**Supplementary Figure 3.** Immune infiltration analysis of NEOLP-S and EOLP-RE samples based on CIBERSORT. Boxplots show significant differences(*p* < 0.05) in immune cells between the NEOLP-S and EOLP-RE groups. The red boxes mark the types of immune cells that are significantly upregulated in the EOLP-RE group.


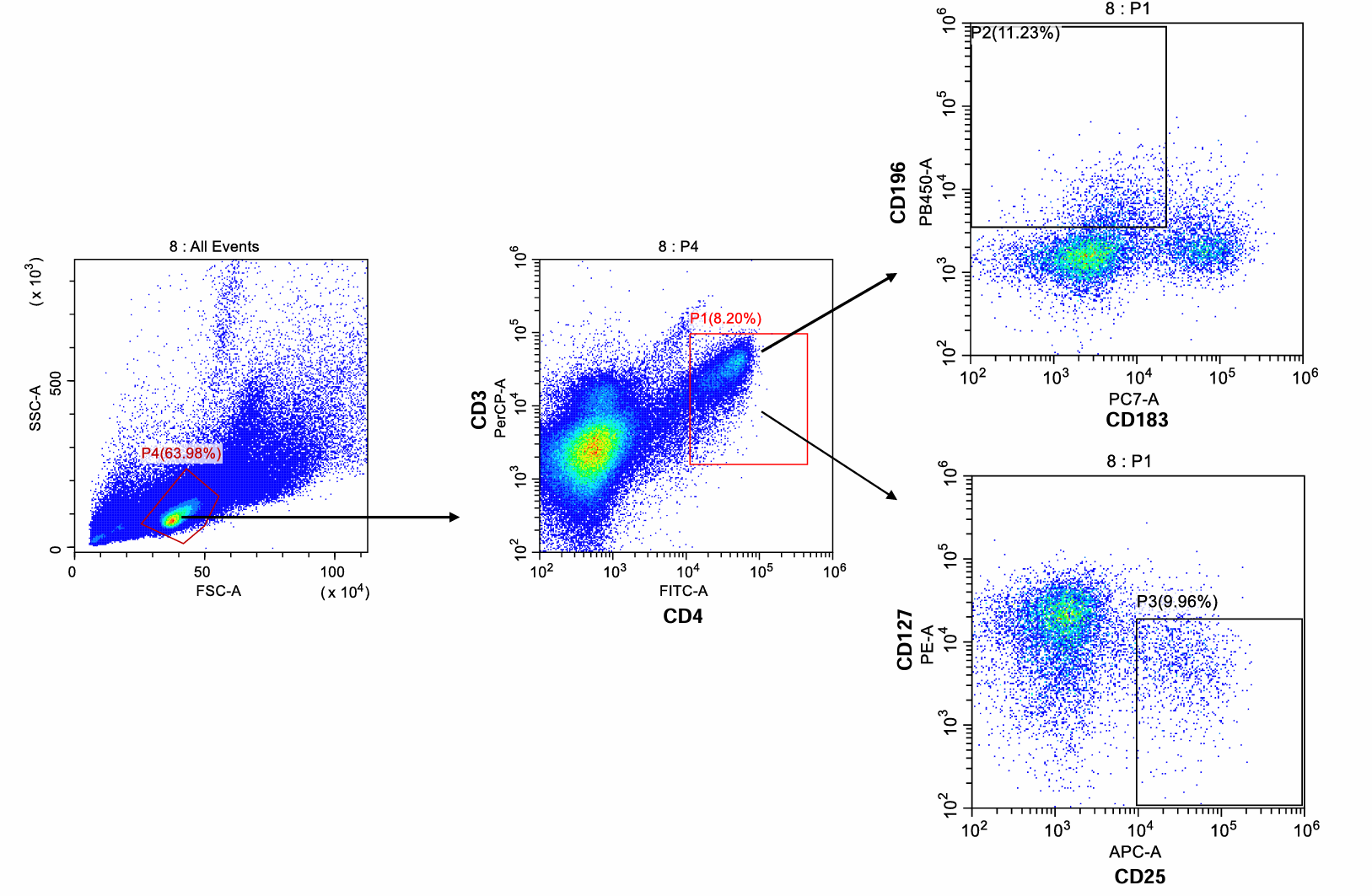


**Supplementary Figure 4.** Flow gating strategies for Th17 cells and Treg cells. After lymphocytes have been delineated, the CD3+ CD4+ cells were labeled as CD4+T cells. Then, the CD3+ CD4+ CD196+ CD183- cells were labeled as Th17 cells, and the CD3+ CD4+ CD25+ CD127- cells were labeled as Treg cells.
